# Protective Effects of a Polyphenol-Rich Blueberry Extract on Adult Human Neural Progenitor Cells

**DOI:** 10.3390/molecules27196152

**Published:** 2022-09-20

**Authors:** Tong Zheng, Donna F. Bielinski, Derek R. Fisher, Jianyi Zhang, Barbara Shukitt-Hale

**Affiliations:** 1 Neuroscience and Aging Laboratory, Jean Mayer USDA Human Nutrition Research Center on Aging, Tufts University, Boston, MA 02111, USA; 2 Friedman School of Nutrition Science and Policy, Tufts University, Boston, MA 02111, USA

**Keywords:** human neural progenitor cells, blueberry, neurogenesis, proliferation, neuroprotection

## Abstract

The aging process impacts neural stem cells and causes a significant decline in neurogenesis that contributes to neuronal dysfunction leading to cognitive decline. Blueberries are rich in polyphenols and have been shown to improve cognition and memory in older humans. While our previous studies have shown that blueberry supplementations can increase neurogenesis in aged rodents, it is not clear whether this finding can be extrapolated to humans. We thus investigated the effects of blueberry treatments on adult hippocampal human neural progenitor cells (AHNPs) that are involved in neurogenesis and potentially in memory and other brain functions. Cultured AHNPs were treated with blueberry extract at different concentrations. Their viability, proliferation, and differentiation were evaluated with and without the presence of a cellular oxidative stressor, dopamine, and potential cellular mechanisms were also investigated. Our data showed that blueberry extract can significantly increase the viability and proliferation rates of control hippocampal AHNPs and can also reverse decreases in viability and proliferation induced by the cellular stressor dopamine. These effects may be associated with blueberry’s anti-inflammatory, antioxidant, and calcium-buffering properties. Polyphenol-rich berry extracts thus confer a neuroprotective effect on human hippocampal progenitor cells in vitro.

## 1. Introduction

Adult neurogenesis is the process by which mammalian neural stem/progenitor cells generate new neurons. It continues throughout the lifespan in the subventricular zone (SVZ) of lateral ventricles and the subgranular zone (SGZ) of the dentate gyrus in the hippocampus [1], and is an essential component of neural plasticity, brain homeostasis, and tissue remodeling of the central nervous system (CNS). There is an established link between preserved hippocampal neurogenesis and healthy cognitive aging [2]. Adult neurogenesis is highly dynamic and is regulated by various internal and external factors [3]. Aging is one of the factors that negatively impact adult neurogenesis [4]. The rate of new neuron generation in the aged brain represents only a small portion of that measured in young adults [5]. Consequently, impaired adult neurogenesis has been reported to impose a direct effect on the age-related decline of brain functions such as cognition and is a hallmark of many neurodegenerative diseases [6]. Even though the adult neural stem cells in aging brains lose their potential for proliferation and become quiescent, it has been suggested that they can be reactivated to a certain degree by lifestyle factors, including diet [7].

The neuropathology of aging and age-related neurodegenerative diseases has been linked to increases in brain oxidative stress, as well as inflammation. Neuroinflammation is characterized by the unregulated activation of microglia and astrocytes, along with elevated levels of circulating cytokines [8]. Inflammation also has been reported to interfere with neuronal stem cell (NSC) function by decreasing NSC proliferation and neuronal differentiation [9], impairing neurogenesis [10] and reducing the survival rate of newly differentiated neurons and their integration into existing neuronal circuits [9,11].

There has been growing interest in the potential neuroprotective effects of plant-derived biocompounds, which have both antioxidant and anti-inflammatory properties. Polyphenols are a class of phytochemicals present in a wide variety of plant foods, such as colorful fruits and vegetables. They are among the most abundant antioxidants and anti-inflammatories in the food supply [12]. Among the many well-studied phytochemical-rich foods are blueberries (BBs), which consist of a mixture of different enriched bioactive, polyphenolic compounds including anthocyanins, flavanols, phenolic acids, and stilbene derivatives, all of which demonstrate health benefits, including anti-inflammatory and antioxidant properties [13]. The beneficial effects of BBs have been largely attributed to their high anthocyanin content [14]. A previous study has shown that several anthocyanins and flavanol metabolites from dietary BB and BB co-digested with grape consumption can cross the blood-brain-barrier and are thus neuroavailable, especially in the hippocampus and cortex, which are involved in cognitive function [15,16]. However, while anthocyanins contribute to the neuroprotective effects of BBs, other polyphenols in BB, such as chlorogenic acid, catechins, and epicatechins, have also demonstrated neuroprotective activities [17,18]. A previous study suggested that the whole BB is more protective than individual polyphenol fractions against stressor-induced CNS deficits, such as stress signal generation and viability of neuronal cells [19]. The superior benefit of the whole fruit may be due to synergistic interactions among blueberry polyphenols or unique properties of individual polyphenols other than anthocyanins [20]. Therefore, the current study focused on the potential effects of whole blueberries instead of a particular polyphenol compound and what mechanisms might be responsible for their beneficial effects.

BBs have been shown to improve cognitive and memory functions in both aged animals [16,21] and humans [22,23]. The putative mechanism(s) underlying blueberry’s protective effects are likely to be multifactorial, including the ability to buffer against excess calcium [24], enhancement of neuroprotective stress shock proteins [25], alteration of inflammatory gene expression, and protection against neurodegeneration following excitotoxic stress [26], and increased neurogenesis [16,21].

While findings in rodent studies regarding the role of blueberry supplementation in increasing neurogenesis are promising, it is unclear whether similar beneficial effects can be extrapolated to human cells or if the mechanisms of action are comparable. Key factors that contribute to the difficulty in replicating and translating current research findings to the human condition include the differences in genetic background between animals and humans [27], the differences between human and rodent neuronal cells [28], as well as the different responses to drug and nutritional supplements between rodents and humans [29]. Specifically, the age-related neurogenesis decline in rodents is more pronounced than in humans [30]. These functional differences may implicate different nutritional requirements between rodents and humans. Because relevant work has been mostly done using animal models, challenges remain to translate existing scientific knowledge acquired from animal studies to human biochemical and physiological processes. To provide a bridge to the gap between animal and human studies, we used human adult neural progenitor cells derived from the hippocampus as an in vitro assay of neurogenesis to address the technical difficulties in accessing human adult neurogenesis and labeling proliferating cells in vivo and to better understand and translate the beneficial effects of blueberries on human neuronal health and function.

Successful use of this model of human neuronal cells does not only help address whether blueberry supplementation would increase neurogenesis, as assessed by viability and proliferation rate, in human neural stem/progenitor cell models but also provides the foundation for important future studies on cellular and molecular mechanisms of actions of other phytonutrients on human neuronal cells.

## 2. Results

### 2.1. Effects of BB on the Viability of AHNPs

**Figure 1:**
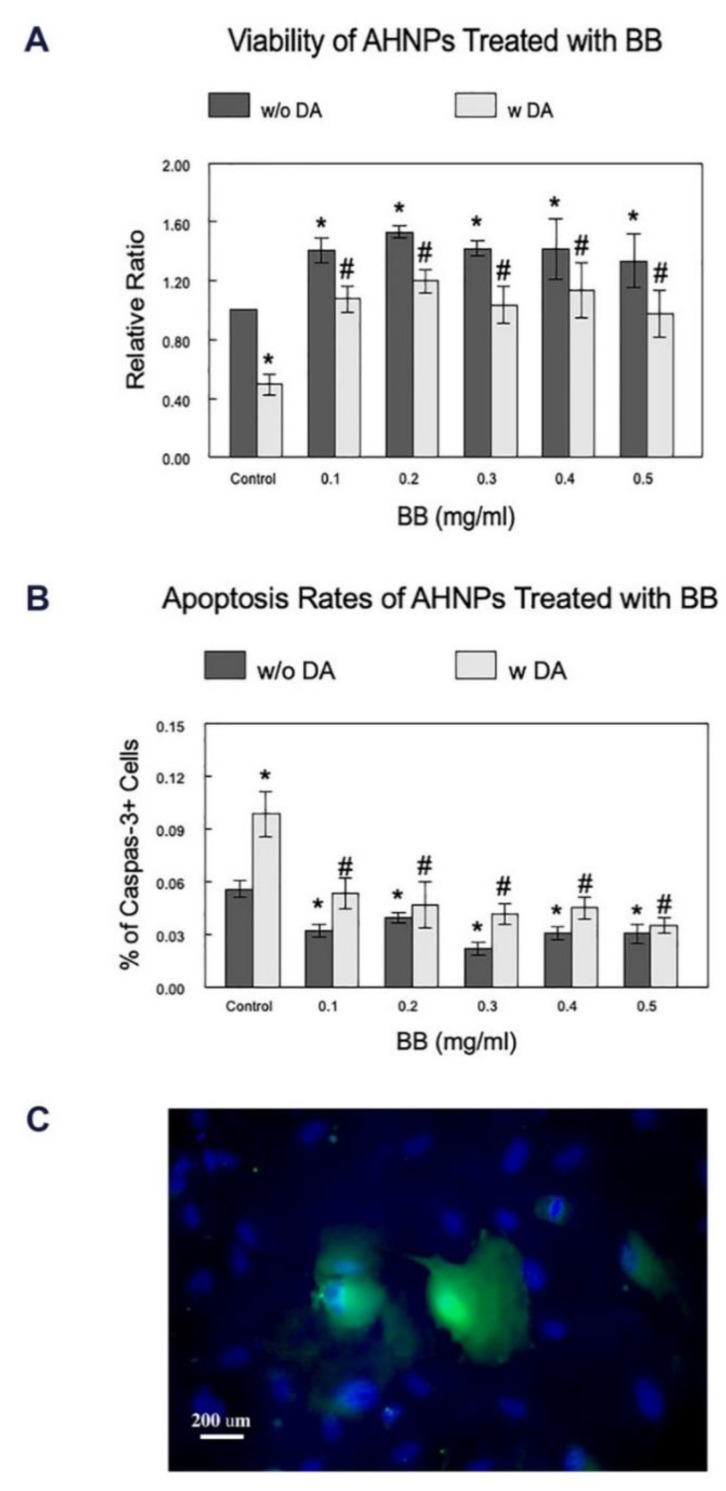
Effect of BB treatment on the viability and apoptosis rates of AHNPs. Viabilities (**A**) and apoptosis rates (**B**) of hippocampal AHNPs treated with blueberry extracts, with or without stressor DA, were evaluated at various concentrations of BB and compared (N = 5 experiments with duplicates of each dosage). Relative ratio in (**A**) was achieved by comparing cell counts of treated cells with those of control cells (without dopamine). The apoptosis of the treated cells was identified using the immunohistochemical labeling of Caspase-3. Example of the Caspase-3 labeling is shown in (**C**) (Green = Caspase-3, Blue = DAPI). Rate of apoptosis (**B**) was achieved by comparing numbers of caspase 3+ cells with the total number of cells labeled with DAPI. Data are presented as mean ± SEM (N = 5 experiments with duplicates of each dosage). * indicates *p* < 0.05 in treated cells vs control w/o DA, # indicates *p* < 0.05 in treated cells vs control with DA.

The viability of AHNPs, either non-stressed or stressed with DA, after pretreatment with BB at various concentrations, was evaluated using the Trypan Blue exclusion assay. As shown in Figure 1A, BB treatment of AHNPs without cellular stressors led to significant increases (*p* < 0.05) in their viabilities compared to the control cells at all tested concentrations (33–53%), suggesting a beneficial effect of BB on the overall viability of the AHNPs. In the presence of the stressor, DA, the viability of the AHNPs was significantly reduced (*p* < 0.01) among cells without BB pre-treatments (50%). However, BB treatments at all tested concentrations significantly increased the viability of the AHNPs reduced due to the cellular stressor, by 96–140%, compared to the stressed cells without BB treatment (*p* < 0.05), therefore protecting against dopamine cytotoxicity. No significant differences were observed among different BB treatment concentrations.

### 2.2. Effects of BB on the Apoptosis Rates of AHNPs

The apoptosis of the treated cells was examined using the immunohistochemical labeling of Caspase-3 (Figure 1B). While activation of caspase-3 is reported to also increase during proliferation and differentiation, the level of increase is significantly higher in apoptotic cells and, thus, is more likely to be detected. Caspase-3 staining remains a reliable and validated tool for assessing apoptosis in neuronal cells and is widely used in the research field. Thus, it is selected as the marker for cell death in this study as a complementary readout to the viability assay described above. Rate of apoptosis was determined by comparing numbers of caspase-3+ cells (Figure 1C, green) with the total number of cells labeled with DAPI (Figure 1C, blue). DA significantly increased the apoptosis rates of the cells among untreated cells by 77% (*p* < 0.0001), and BB treatment was able to significantly attenuate (*p* < 0.05) the stress-induced increase of apoptosis rate of AHNPs at all tested concentrations by 45–65% (Figure 1B). Our data also indicated that BB treatment could significantly reduce (*p* < 0.05) the apoptosis rate of non-stressed cells at all tested concentrations by 29–61%, confirming the beneficial effect of BB against general cell loss due to apoptosis.

### 2.3. Effects of BB on the Proliferation of AHNPs

**Figure 2:**
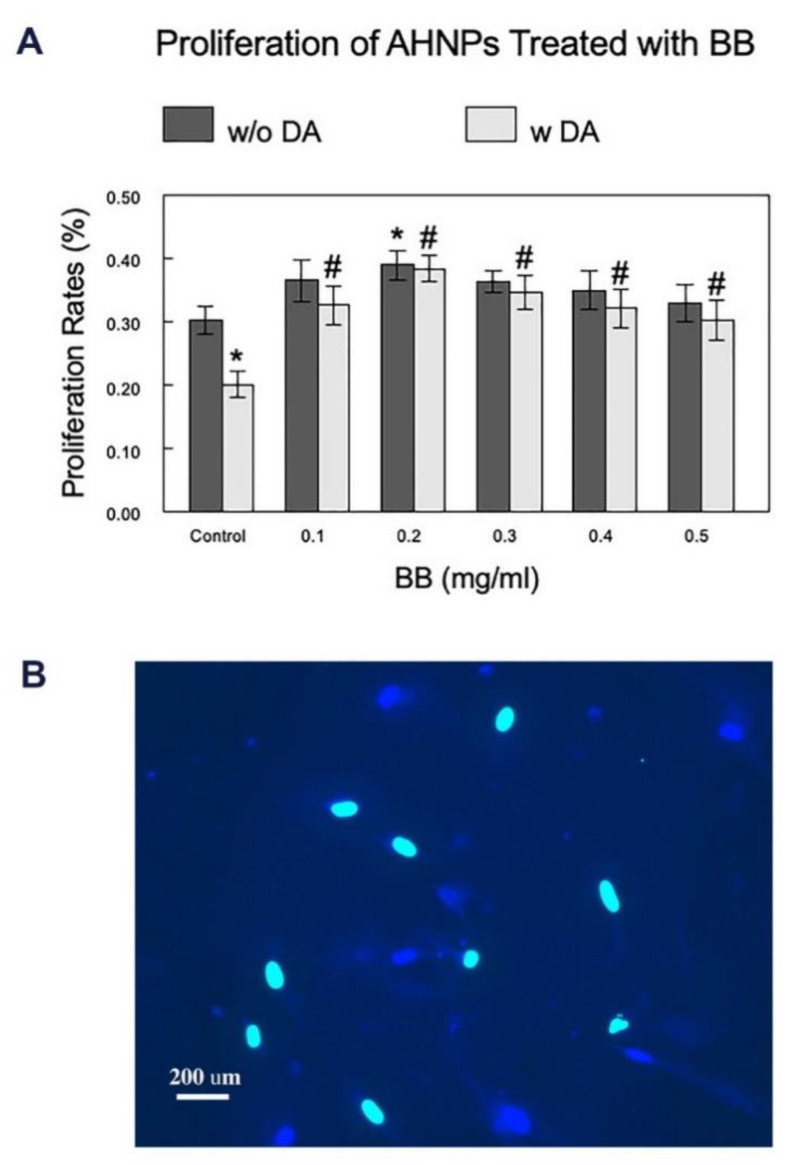
Effects of BB on the proliferation of AHNPs. Proliferation rates of hippocampal AHNPs treated with blueberry extract, with or without stressor DA, were evaluated at various concentrations of BB and compared (**A**). Proliferation of AHNPs was identified using the EdU assay, with a representative image shown in (**B**) (Green = EDU+, Blue = DAPI). Percentages of newly generated cells were achieved by comparing the EDU+ cells and the total number of cells labeled with DAPI. Data are presented as mean ± SEM (N = 5 experiments with duplicates of each dosage). * indicates *p* < 0.05 in treated cells vs control w/o DA, # indicates *p* < 0.05 in treated cells vs control with DA.

Percentages of newly generated cells (Figure 2A) were determined by comparing the EdU+ cells (Figure 2B, green) and the total number of cells that were labeled with Hoechst 33342 (Figure 2B, blue). As shown in Figure 2A, BB treatment in AHNPs without cellular stressors resulted in a significant increase in proliferation rate (30%), compared to the control cells, but only at a dose of 0.2 mg/mL (*p* < 0.05). However, similar to what was observed in viability assays, in the presence of DA, the proliferation rate was significantly reduced (*p* < 0.001) in the stressed control cells by 33%, and pretreatments with BB at all tested concentrations were able to significantly attenuate (*p* < 0.05) the DA-induced decrease in the proliferation rate by 50–90%, which indicates the potential neuroprotective effect of BB on the neurogenic capacity of the AHNPs.

### 2.4. Effects of BB on the Phenotyping of AHNPs

**Figure 3:**
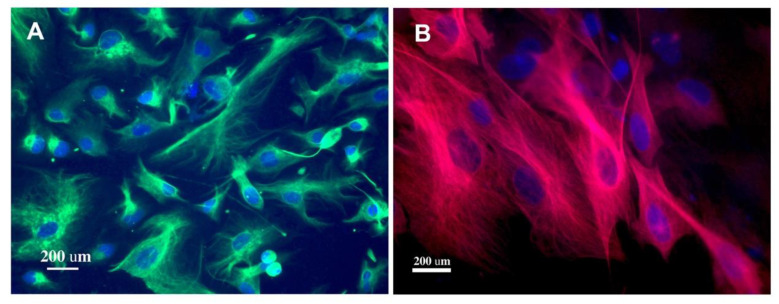
Effects of BB on the phenotyping of AHNPs. Highly expandable AHNPs ubiquitously expressed the neural stem/progenitor cell marker Nestin (**A**). The majority of them also expressed the astrocyte marker GFAP (**B**). Treating the cells with BB extracts did not affect the cellular phenotypes of the AHNPs.

Treating the cells with BB (with and without DA) did not detectably alter the phenotypes of the AHNPs, as they continued to be wholly positive for Nestin (Figure 3A), with the majority of them also expressing the astrocyte marker GFAP (Figure 3B). This finding is consistent with a previous report on the characterization of AHNPs [31], which showed they are predominantly astrocytes (GFAP+) with neurogenic properties (Nestin+).

### 2.5. Effects of BB on the Calcium Buffering Ability of AHNPs

Potential cellular mechanisms underlying the beneficial effects of the BB on the viability and proliferation of AHNPs were investigated using cells treated with 0.1 mg/mL BB extract, the lowest concentration that demonstrated effects. Because at least part of the loss of cognitive function in aging may be dependent upon a dysregulation in calcium homeostasis, and because this loss affects numerous signaling pathways, we investigated whether BB could enhance calcium buffering and/or reduce stress signaling. Using Fura-2 AM dye to bind to intracellular calcium, the percent of cells in which the Ca^2+^ level returned to 30% of the increase following depolarization (% Cells recovered), and the time taken to return to 30% of the Ca^2+^ increase in those cells that recovered (time to recover), were determined in the AHNP cells with and without pretreatment of BB, followed by DA-induced stress.

**Figure 4:**
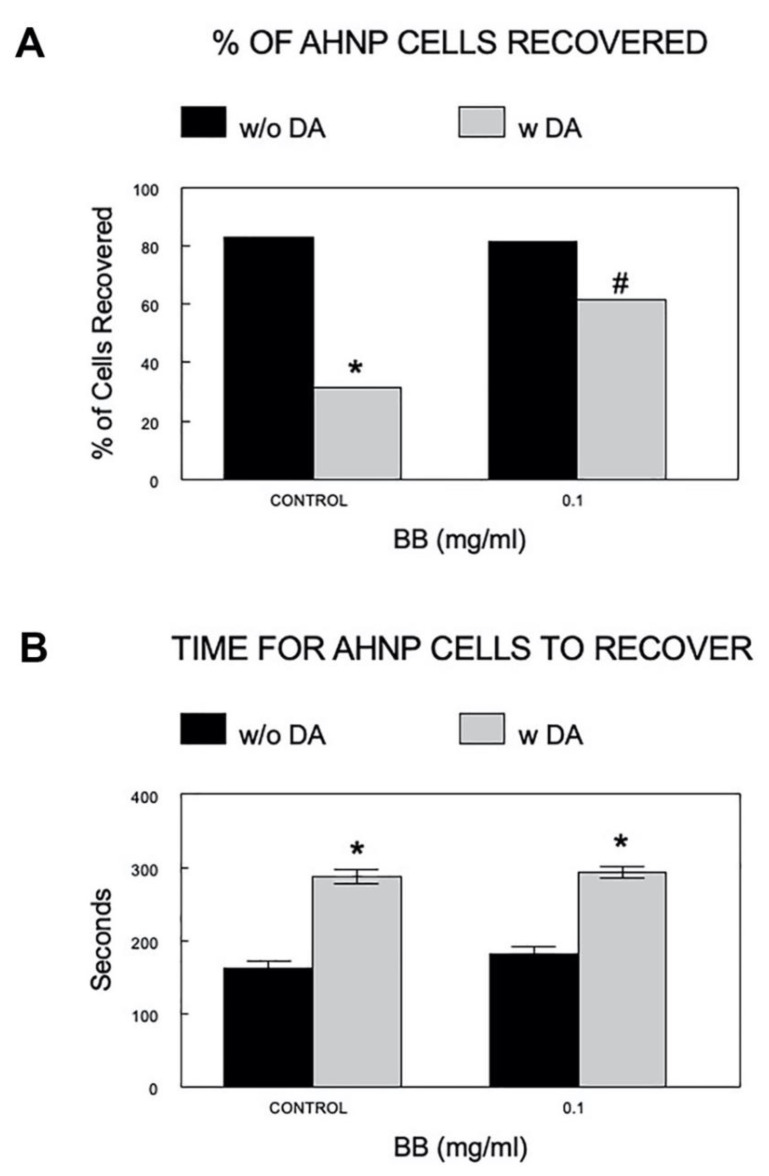
Effects of BB on the calcium buffering ability of AHNPs. Using Fura-2 AM dye to bind to intracellular calcium, the percent of cells in which the Ca^2+^ level returned to 30% of the increase following depolarization ((**A**): % Cells Recovered), and the time taken to return to 30% of the Ca^2+^ increase in those cells that recovered ((**B**): Time to Recover) were determined in the hippocampal AHNPs with and without pretreatment of BB (0.1 mg/mL), followed by DA-induced stress. Data are presented as mean ± SEM (N = 4 experiments with duplicates of each dosage). * indicates *p* < 0.05 vs control w/o DA, # indicates *p* < 0.05 vs control with DA.

As shown in Figure 4A, administration of DA significantly reduced the percent recovery of AHNP cells, and BB extract (0.1 mg/mL) was able to protect against the DA-induced decreases in percent recovery of cells (*p* < 0.05). There was a significant increase in the time needed for the AHNPs to recover from DA (*p* < 0.001). BB treatment did not significantly reduce the prolonged recovery time induced by DA in AHNPs (Figure 4B).

### 2.6. Effects of BB on Expression of Oxidative and Inflammatory Markers of the AHNPs

**Figure 5:**
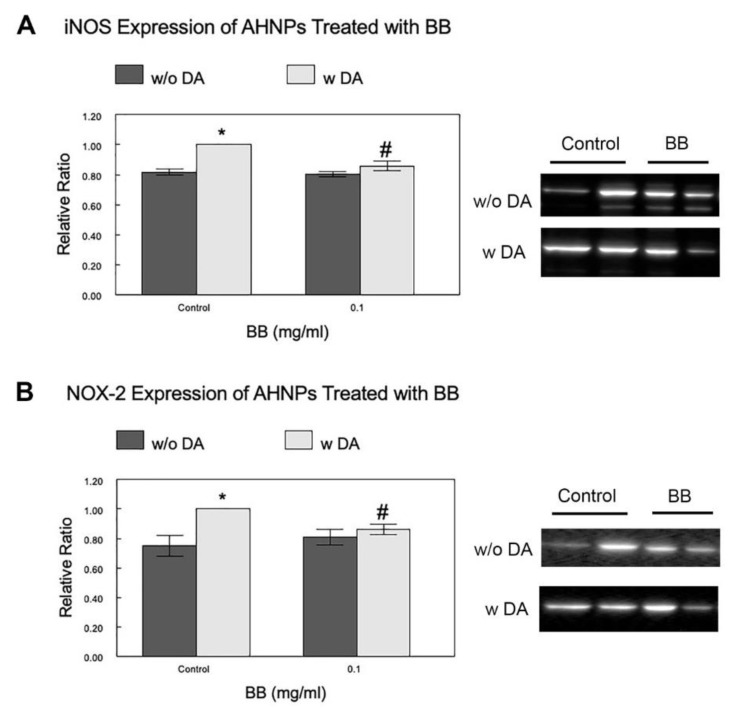
Effects of BB on expression of oxidative and INF markers of the AHNPs. The expressions of inflammation and oxidative stress markers, iNOS (**A**) and NOX-2 (**B**), of AHNPs, treated with blueberry extracts (0.1 mg/mL), with or without stressor DA, were evaluated using western blot analysis of cell lysates. Relative ratio was achieved by comparing the mean protein band densities of treated cells with those of the control cells stressed with DA. Example western blot images of both markers are shown on the right. Data are presented as mean + SEM (N = 4 experiments with duplicates of each dosage). * indicates *p* < 0.05 vs control w/o DA, # indicates *p* < 0.05 in treated cells vs control with DA.

To further explore the mechanisms of BBs’ beneficial effects, the expression of oxidative stress marker NOX-2, and inflammation marker iNOS, in AHNPs treated with blueberry extracts (0.1 mg/mL), with or without stressor DA, was evaluated using western blot analyses of cell lysates. The relative ratio was achieved by comparing the mean protein band densities of treated cells with those of the control cells stressed with DA. As shown in Figure 5, our data indicate that DA-induced stress significantly increased (*p* < 0.05) the expression level of iNOS by 22% (Figure 5A) and NOX-2 by 33% (Figure 5B) in control hippocampal AHNPs, and that the blueberry treatments (0.1 mg/mL) significantly attenuated (*p* < 0.05) the DA-induced increased expressions of both iNOS and NOX-2 by 14%.

## 3. Materials and Methods

### 3.1. Cell Culture

Adult human neural progenitor cells (AHNPs) were used in an in vitro assay to evaluate the effects of the BB treatments. AHNPs were developed, isolated, and characterized as previously described [31]. For the current study, AHNPs previously isolated from human hippocampal biopsy samples were cultured in uncoated tissue culture flasks in proliferation media consisting of Dulbecco’s modified Eagle’s medium (DMEM, Invitrogen, 11320033, Waltham, MA, USA), N2 supplement (Invitrogen, 17502048), antibiotics (Invitrogen, 15240062), and supplemented with 35 µg/mL bovine pituitary extract (Invitrogen, 13028014), 5% fetal bovine serum (FBS, Atlanta biological, S11150, Flowery Branch, GA, USA), 40 ng/mL epidermal growth factor (EGF, Sigma, E9644, St. Louis, MO, USA), and 20 ng/mL basic fibroblast growth factor (bFGF, Sigma, F0291). The media was changed every 3 days, and confluent cells were passaged using Trypsin and split 1:3 for expansion. For follow-up analyses, confluent AHNPs were plated on uncoated multi-well culture plates or chamber slides at an appropriate density, according to each experimental setting. They were then treated with blueberry extract, as detailed below.

### 3.2. Blueberry Extract Preparation and Treatments

The blueberry (BB) powder was prepared by homogenizing Tifblue blueberries (provided by the US Highbush Blueberry Council, Folsom, CA, USA) with deionized water (1:1 *w*/*v*) for 3 min and then centrifuging the recovered homogenate at 27,500 g for 15 min at 4 °C. The supernatant was then frozen, crushed, lyophilized, and characterized, as previously described [32], and the resulting BB powder had a phenolic level of ~10.15 mg/g [19]. Based on our preliminary experiments, concentrations of blueberry extract that ranged between 0.1 to 0.5 mg/mL showed effects on non-stressed cells without being cytotoxic and therefore were chosen to be the concentrations for this study. The BB powder was dissolved in growth media and added to the cells (0–0.5 mg/mL), which were subsequently incubated for 7 days starting the day after the cells were plated. The media was changed with fresh extract every 48 h. The cells were washed three times with extract-free growth medium at the end of the treatment period, prior to follow-up evaluations.

### 3.3. Cellular Stressor Treatments

To determine whether BB extract can protect AHNPs from oxidative or inflammatory stress, a subpopulation of AHNPs was treated with a cellular stressor, dopamine (DA, Sigma, H8502, 0.1 mM), for 4 h at the end of BB treatments. DA was used as an oxidative stressor in the current study because it rapidly oxidizes to form reactive oxygen species and quinones, and its oxidation may be involved in neuronal toxicity in neurodegenerative disease [19,33]. Stressed cells with or without BB treatments were then subjected to assessments of viability, differentiation, and proliferation rate, as described below.

### 3.4. Cell Viability Assay

The viability of the treated cells was examined using the Trypan Blue exclusion method [34]. In brief, an equal number of AHNPs for each well were seeded on uncoated 6-well plates. They were then treated with BB extract of various concentrations, as described above. Cells were then collected and suspended into single-cell suspensions. A 0.4% solution of Trypan Blue (Sigma, T8145) was added to an aliquot of cell suspension at a ratio of 1:1. The cells were then examined and counted immediately with a hemacytometer under a microscope. The number of blue-stained cells and total cells were counted, and the percentage of viable cells was calculated. The results are shown as the percent viability of the treated groups relative to the control (untreated; non-stressed cells without BB treatment), which was considered 100%.

Because the reduction of neural stem cell numbers during aging and neurodegeneration is related to apoptosis, which is cell death through a protease enzyme family caspase, the apoptosis rate of control and BB-treated AHNPs, with and without DA stressor, was evaluated using immunofluorescent labeling against a Caspase-3 antibody (Abcam, ab13847, Cambridge, UK), as described below. The rate of apoptosis was achieved by comparing the numbers of caspase-3+ cells with the total number of cells.

### 3.5. Proliferation Assay

EdU (5-ethynyl-2′-deoxyuridine), a nucleoside analog of thymidine that can be incorporated into DNA during active DNA synthesis, was added to the culture media at a concentration of 10 µM, starting with the first BB treatment, and supplemented whenever fresh media and BB treatments were added. On the last day of blueberry treatment, a Click-iT Edu Imaging Kit (Invitrogen, 10637) was used to visualize and measure cell proliferation [35]. Briefly, cells plated on coverslips were washed with PBS and fixed with 4% paraformaldehyde. They were then permeabilized with 0.1% Tween-20 for 20 min, followed by the detection of EdU according to the manufacturer’s protocol. EdU labeling was combined with staining of nuclei with DAPI or Hoechst 33342 (5 µg/mL). The number of EdU-labeled cells was compared with the total cell number to determine the cell proliferation rate.

### 3.6. Calcium (Ca^2+^) Imaging

Calcium buffering capacity was assessed by using imaging procedures as described by Joseph et al. [19]. After the BB treatments, cells were loaded with cell-permeable Fura-2 AM dye (Fura2/acetoxymethyl ester, 2 µM) in loading medium (99% DMEM, 1% FBS) for 40 min at 37 °C with 5% CO_2_. The Fura-2 AM binds to free intracellular calcium, and the cells were then depolarized by a 20-min incubation in KRH buffer with 30 mM KCl to induce calcium (Ca^2+^) entry into the cells. Imaging was conducted using a Nikon TE2000-U inverted microscope illuminated with a fluorescent light source. Percent cells recovered and recovery time, the amount of time required for the Ca^2+^ level to return to 30% of the increase following depolarization, were determined.

### 3.7. Phenotyping

To examine whether BB extract affects the cellular characteristics of the treated AHNPs, a randomly selected portion of the cells was cultured on laminin/polyornithine-coated coverslips [31], followed by the treatments. The cells were then fixed with 4% paraformaldehyde on the last day of treatment and further processed for immunohistochemical labeling. The cells were blocked in PBS supplemented with 0.1% Tween-20 (PBSt) and 10% FBS for 1 h at 37 °C, followed by incubation in primary antibodies against an astrocyte-specific marker (GFAP, 1:1000, Dako, Z0334, Santa Clara, CA, USA), as well as a neural stem/progenitor cell marker (Nestin, 1:1000, Millipore, MAB353, Burlington, MA, USA), overnight at 4 °C. Cells were then washed in PBSt before the addition of fluorescence-labeled secondary antibodies followed by mounting and coverslipping with Vectashield mounting media containing DAPI (Vector Lab, H1200, Newark, CA, USA). Labeled cells were evaluated using an epifluorescence microscope.

### 3.8. Western Blot

To assess BB effects on oxidative stress and inflammation, western blot techniques were used to examine the expression levels of NADPH oxidase 2 (NOX-2), and Inducible nitric oxide synthase (iNOS) as previously described [36]. These markers were selected because iNOS, as one of the key enzymes generating nitric oxide (NO), plays an important role in numerous neural, physiological, and pathophysiological conditions, e.g., neuroinflammation. NOX-2, a super-oxide generating enzyme that forms reactive oxygen species (ROS), is highly expressed in cells throughout the CNS and is a major generator of ROS in many neuropathological conditions. It is also suggested to be linked to hippocampal NSC proliferation and neuronal commitment [37].

At the end of treatments, cells were washed in ice-cold PBS, re-suspended, and lysed by agitation and scraping in CelLytic-M Cell Lysis Reagent (Sigma), to which protease inhibitors were added. Cells were then centrifuged at 18,000 *g* for 10 min at 4 °C. The resultant supernatant lysate was used for blotting, and the pellet was discarded. Western blots were performed by running 20 µg total protein per lane on 10% polyacrylamide gels before transfer onto PVDF membranes. Primary antibodies for iNOS (Millipore, ABN26) and NOX-2 (Santa Cruz Biotechnology, sc-130543, Dallas, TX, USA) were used at 1:1000 dilutions for incubation overnight at 4 °C, followed by secondary antibody incubation. The signal was detected using an electrochemiluminescence (ECL) detection kit (BioRad, Hercules, CA, USA), and the optical density of antibody-specific bands was analyzed by the VisionWorks LS image acquisition and analysis 8.1.2 software (UVP, Upland, CA, USA). Values were normalized to GAPDH protein levels.

### 3.9. Statistical Analyses

The results were expressed as mean ± SEM from a minimum of four experiments; treatments were performed in duplicate for each experiment. For viability, proliferation, and western blot analysis, two-way analyses of variance (ANOVA) with BB treatment concentrations and stress exposure as experimental factors were conducted, using a general linear model in SAS 9.4, followed by a post hoc pair-wise *t*-test for multiple comparisons. For calcium image analyses, percent recovery of the cells was analyzed by Kruskal-Wallis one-way ANOVA and Mann–Whitney U post hoc tests, while time to recover was analyzed by ANOVA followed by post hoc testing with Fisher’s LSD test. Image J 1.53 software from NIH was used for cell counts. All results were considered significant at *p* < 0.05.

## 4. Discussion

The primary outcome of this study demonstrated that the adult human neural progenitor cells are sensitive to cellular stress, which showed negative impacts on their survival and proliferation. Blueberry extract pretreatment was able to attenuate these negative impacts without changing their phenotypes, therefore suggesting a potential role of blueberries in the protection of adult human neural progenitor cells. The secondary outcomes of the study suggested that the underlying cellular mechanism of BBs’ protective effects may be related to their calcium buffering ability, as well as their anti-inflammatory and antioxidant properties.

Reports from both our lab and other labs have demonstrated the beneficial effects of BB on rodent CNS in both in vivo and in vitro models. Specifically, BB has been shown to increase the proliferation of hippocampal precursor cells in BB-fed aged rats [21] and to increase neuroplasticity in aged mice fed with a high-fat diet [38]. A combination of polyphenol-rich grape and BB extracts was able to increase the proportion of newly generated neurons with prolonged dendrites in aged mice, suggesting BB’s effects on neuronal differentiation and maturation [16]. However, studies on the effects of BB and other nutrients on human neural stem cells remain very limited. The hippocampal AHNPs used in this study were previously characterized and demonstrated an ability to generate diverse neuronal populations [31,39]. These cells represent the indigenous cells responsible for neurogenesis and can be utilized as an in vitro bioassay for testing diet and phytonutrient components that have potential beneficial bioactions on human neurogenesis. Such an assay allows us to address technical difficulties in translating existing scientific knowledge acquired from animal cells to human cells and in investigating human neurogenesis in vivo.

During aging and stress, reduced numbers of neural stem cells cause impaired neurogenesis and contribute to neurodegeneration [40]. We demonstrated here that BB treatments had beneficial effects on the overall survival of the AHNPs and, furthermore, could attenuate the decrease in viability of the AHNPs induced by a cellular stressor. Additionally, blueberry extract decreased the percentage of apoptotic AHNPs, both stressed and non-stressed. These observations support the hypothesis that the reduction of stem cell numbers due to stress and during neurodegeneration may be related to apoptosis [41]. Similar protective effects of BB extract were reported on the viability of rodent hippocampal neurons stressed by H_2_O_2_ and dopamine [19,42]. These findings indicated that BB extract might play a neuroprotective role in helping to prevent neural cell loss. Future studies are required to determine if these observations are consistent with changes associated with aging.

In addition to the decrease in the survival of neural stem cells, one of the most notifiable impacts of aging, stress, and neurodegeneration on adult neural stem cells is the progressive reduction in proliferation [5]. Similar to a previous study using human hematopoietic stem cells [43], we showed that BB was able to reverse the decrease in proliferation of human neural stem/progenitor cells induced by a cellular stressor. Decreased hippocampal neurogenesis due to reduced proliferation is an important mechanism underlying age-related cognitive decline as well as neurodegeneration and various types of dementia [44]. Therefore, our finding that BB had beneficial effects on the proliferation of human hippocampal neural stem cells, especially on the rescue of the stress-induced decline of proliferation, supports its potential neuroprotective actions on human neurogenesis.

Our data, both previously in rodent cells as well as here on human hippocampal progenitor cells, demonstrate the beneficial effects of BB on the survival and proliferation of neural stem cells. However, the mechanisms of such effects remain to be determined. It is hypothesized that rather than one overriding mechanism, multiple underlying mechanisms could be present, and this study examined two possible mechanisms. Calcium buffering ability was considered one of the mechanisms because Ca^2+^ is an important second messenger that is involved in a wide range of cellular functions, including proliferation [45], and sustained increases of intracellular Ca^2+^ due to the loss of calcium buffering ability increase the sensitivity of the neural progenitor cells to stress and may lead to cell death and reduced cell proliferation [46]. Our data showed that the cellular stressor dopamine reduced the percentage of AHNPs that could recover from sustained Ca^2+^ influx by increasing the time the cells took to recover. However, BB extract showed protective effects by increasing the percentage of recovered cells but not by decreasing the recovery time. Therefore, BB treatment may help preserve part of the calcium buffering ability of the AHNPs and thus contribute to the protection of their survival and proliferation under stress.

The other possible mechanisms underlying the protective effects we observed in this study may be related to the well-known anti-inflammatory and antioxidant properties of BB, as neuroinflammation and oxidative stress have been reported to interfere with neuronal stem cell function, including proliferation [9]. We found that BB extract significantly reduced the expression of the inflammatory marker iNOS, as well as the expression of the oxidative stress marker NOX-2, in cells stressed with DA. These data suggest that BB’s effects on AHNPs may be partly due to its ability to inhibit inflammatory and oxidative pathways. Given the fact that an association was found between oxidative stress/neuroinflammation and age-impacted neurogenesis/cognitive performance in rodents [21], the berry-mediated effects on inflammation and oxidative stress may play a role in improving age-related cognitive functions as well. Regardless of the mechanism, we did not observe a dose effect of BB on the survival and proliferation of hippocampal AHNPs. This lack of dose dependence could be due to the lowest BB concentration we tested (0.1 mg/mL) being sufficient to trigger the protective effects and to the range of concentrations we tested (0.1–0.5 mg/mL) not being high enough to produce further differences.

Additionally, the protected effects we observed were more apparent in the cells under dopamine-induced stress. This phenomenon was also reported in rodent microglia cells challenged with lipopolysaccharide (LPS) [47] and in animals stressed with kainic acid [48], suggesting the role of BB in the prevention of inflammatory insults and cellular stress. This finding is especially important as adult neural stem/progenitor cells are known to be particularly sensitive to stress [49] and neuroinflammation [50], which, in turn, could affect the level of hippocampal neurogenesis. The current study, as well as the majority of previously published studies, examined the beneficial effects of BBs on their preventative (i.e., pre-stressor) role in neuroinflammation, oxidative stress, and/or cognitive function. However, post-treatments may be necessary to repair damage from the initial stress response and prevent the active progression of neurodegeneration long-term. A recent study applying BB both before and after cellular stressor on rodent microglial cells showed significantly reduced neuroinflammatory markers, suggesting BB’s therapeutic effects after the initiation of the cellular stress [51].

This study provides novel information on the beneficial effects of BB directly on human cells using hippocampal neural progenitors in an in vitro assay. However, we do acknowledge the limitations of using an in vitro model, as these models are not a complete realistic replication of physiological conditions and may lead to inaccurate interpretations. Nevertheless, with consideration of the profound functional and genetic difference between rodents and humans, and the technical difficulties in accessing human adult neurogenesis, we believe that human cell assays may provide useful and quick methods for screening the effects of bioactives on those cells and provide tools for investigating mechanisms behind the protective actions. Future studies using multiple models are required to bridge the gap between in vitro, animal, and human studies.

In conclusion, the current study evaluated the impact of blueberries on human adult hippocampal neural stem progenitor cells, which decline during aging and neurodegenerative diseases. We demonstrated that BB extract has beneficial effects on the survival and proliferation of hippocampal neural stem progenitor cells, which may be related to BB’s anti-inflammatory and antioxidant properties and its ability to maintain intracellular calcium homeostasis. Our findings support the notion that dietary intervention using food-derived nutrients or whole foods, such as blueberry, could protect neurogenesis from the cellular stresses that lead to the detrimental effects of aging on the brain.

## Data Availability

All data are available upon request.

## Abbreviations

AHNPs	Adult hippocampal human neural progenitor cells
ANOVA	Analyses of variance
BBs	Blueberries
bFGF	Basic fibroblast growth factor
Ca^2+^	Calcium
CNS	Central nervous system
DA	Dopamine
DMEM	Dulbecco’s modified Eagle’s medium
ECL	Electrochemiluminescence
EdU	5-ethynyl-2′-deoxyuridine
EGF	Epidermal growth factor
FBS	Fetal bovine serum
iNOS	Inducible nitric oxide synthase
LPS	Lipopolysaccharide
NOX-2	NADPH oxidase 2
NSC	Neuronal stem cell
PBSt	PBS supplemented with Tween-20
SGZ	Subgranular zone
SVZ	Subventricular zone
